# Treatment Alternatives for Recurrent Esophagogastric Junction Adenocarcinoma: Case Report of an Ileocolonic Reconstruction and Literature Review

**DOI:** 10.7759/cureus.9504

**Published:** 2020-08-01

**Authors:** Juan Javier Acevedo, Juliana Restrepo, Dary Hernandez, Ricardo Oliveros, Raúl Pinilla

**Affiliations:** 1 Gastrointestinal Surgery, Instituto Nacional De Cancerología, Universidad Militar Nueva Granada, Bogota, COL; 2 Surgical Oncology, Instituto Nacional de Cancerología, Bogota, COL; 3 Surgical Oncology, Universidad Militar Nueva Granada, Bogota, COL; 4 Oncologic Surgery, Universidad Militar Nueva Granada, Bogota, COL; 5 Gastrointestinal Surgery, Instituto Nacional de Cancerología, Bogota, COL

**Keywords:** esophagogastric junction, adenocarcinoma, esophagectomy, reconstructive surgical procedures, colon

## Abstract

Esophagogastric junction tumors are a challenging pathology for surgeons and the best treatment depends on an adequate initial localization and stadification. Approximately half of patients relapse after curative surgery during the first two years. Surgical resection could increase the survival of these patients, but the esophageal reconstruction is a surgical challenge for which there are multiple reconstruction techniques described with different organs.

In this report, we present the case of a patient with an esophagogastric junction tumor treated initially with total gastrectomy and esophageal margin. The patient presented an anastomotic recurrence that was taken to surgical resection, but a second recurrence required a residual esophagectomy with ileocolonic reconstruction, to achieve adequate oncologic treatment.

## Introduction

Despite multiple therapeutic modalities, esophagogastric junction (EGJ) tumors have a poor prognosis, with a five-year survival rate of 8% in 1973 and an increase to 17% in 2008, due to the introduction of neoadjuvant chemoradiotherapy [1]. An initial endoscopic study is the cornerstone to diagnose and locate the tumor, essential elements in defining the appropriate treatment.

Recurrence of these tumors after surgical resection with curative intent is around 40% and many occur in the first two years. Surgical rescue is still controversial, as some studies show the limited role of surgery, while others report increased survival of patients undergoing surgical rescue [2].

There are several reconstruction alternatives after esophagectomy when the stomach is not available, and the decision is made based on the replacement organ, its length, and its vasculature [3]. The gastrointestinal surgeon and the surgical oncologist must be familiar with all these alternatives when treating this type of patients.

## Case presentation

**Figure 1:**
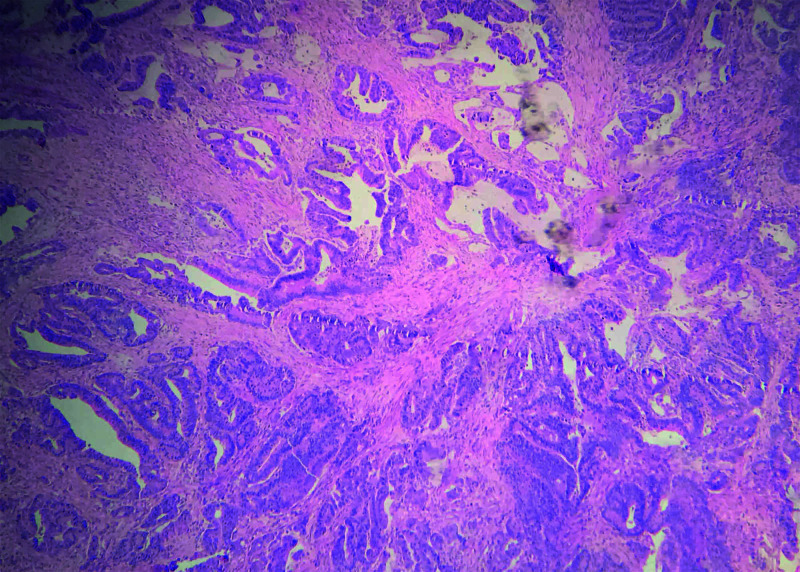
Gastric compromise due to well-differentiated adenocarcinoma

A 55-year-old man with a relevant history of back injury from a multiple-load firearm projectile consulted the Instituto Nacional de Cancerología (INC) in November 2015 with a three-month history of dysphagia, vomiting and 20 kilograms (kg) of weight loss. Studies were performed, and upper gastrointestinal endoscopy (UGIE) showed a tumor lesion in the EGJ with obstruction, which compromised the entire esophageal circumference from 39 to 44 cm. In this endoscopic study, the Z line was compromised by a tumor, not allowing an adequate definition of its localization. It was deduced that it could be 41 cm from the dental arch, with no findings of compromise in the fundus or gastric body, and was classified as a Siewert type II. Pathology reports indicated a well-differentiated adenocarcinoma (Figure 1), and an abdominal CT scan showed thickening of the EGJ with signs of compromise to the serosa and a retroperitoneal node at the upper limit of normal size. A CT chest scan reported multiple metallic-density foreign bodies corresponding to multi-loading firearm projectiles and dilatation of both the thoracic and cervical esophagus by the mass described in the EGJ.

**Figure 2:**
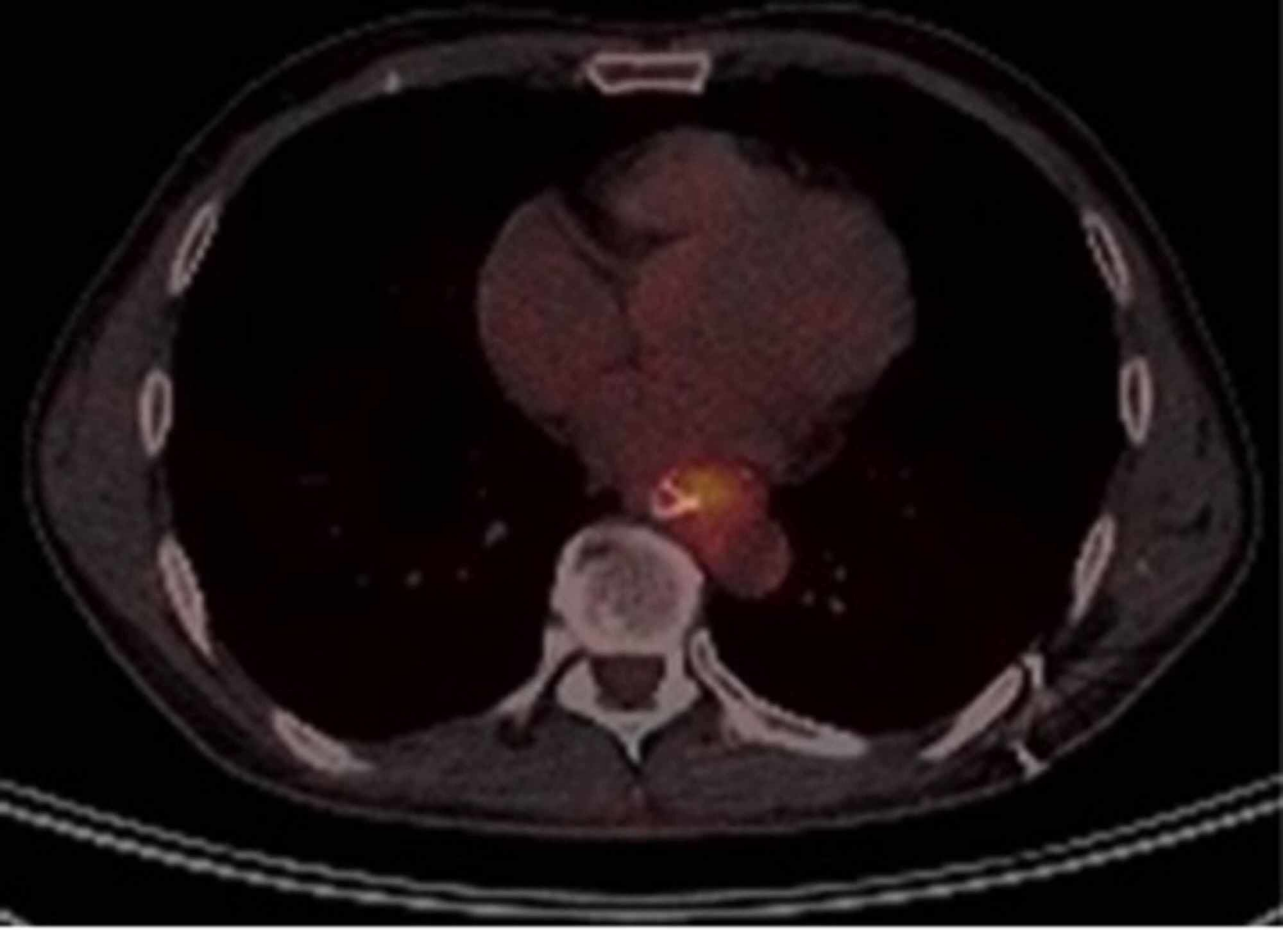
PET-CT with hypermetabolism in the esophagus-jejunal anastomosis due to relapse of a known primary tumor

Due to these findings, it was decided to use a self-expanding metal prosthesis, and in a multidisciplinary meeting, it was defined that the best therapeutic option for this patient with locally advanced EGJ adenocarcinoma was neoadjuvant chemo-radiotherapy with paclitaxel plus carboplatin, concomitant with radiotherapy 50.4 Gy for five weeks (CROSS). In March 2016, restaging was carried out, which showed infiltration of the fundus and minor curvature of the gastric body. Surgery was opted for, a total trans-abdominal gastrectomy plus distal esophagectomy with modified D2 drainage. Surgical pathology reported a well-differentiated intestinal-type adenocarcinoma with poor response to neoadjuvant therapy, positive proximal resection margin for adenocarcinoma, but a negative esophageal donut, and four of 18 lymph node-positive metastases. Pathological staging was ypT3ypN2 for stage IIIA according to TNM 8th ed. After surgical resection, the patient received chemotherapy with cisplatin plus capecitabine (ARTIST) without radiotherapy. During follow-up, elevation of the carcinoembryonic antigen (CEA) was documented, and in a new UGIE in April 2017 a 3-mm nodule was identified in the esophageal-jejunal anastomosis whose pathology result was positive for adenocarcinoma recurrence. A positron emission tomography (PET-CT) was performed, which reported hypermetabolism at the esophagus-jejunal junction with an SUV max of 3.7 (Figure 2) without distant disease, so it was decided to return to surgery for surgical rescue.

In August 2017, resection of the esophagus-jejunal anastomosis and reconstruction with a jejunal loop were performed via transhiatal abdominal approach. The pathology result of the resection reported compromise with a moderately differentiated infiltrating intestinal-type adenocarcinoma with free edges and perineural and lymphovascular invasion, in addition to one of four lymph nodes affected by the tumor. During the oncology meeting it was concluded there was no evidence of the usefulness of additional systemic treatment, and follow-up with the patient continued. In February 2018, the patient presented an elevated CEA again, so a PET-CT was performed that reported an increase in metabolic activity at the site of the last esophageal-jejunal anastomosis, but the UGIE did not show endoluminal tumor relapse and the decision was taken to continue with expectant management. In May 2018, the patient continued to have elevated CEA and a new UGIE identified a nodular mucosa and anastomosis with compromise of 30% of the circumference, and biopsies reported compromise due to adenocarcinoma.

**Figure 3:**
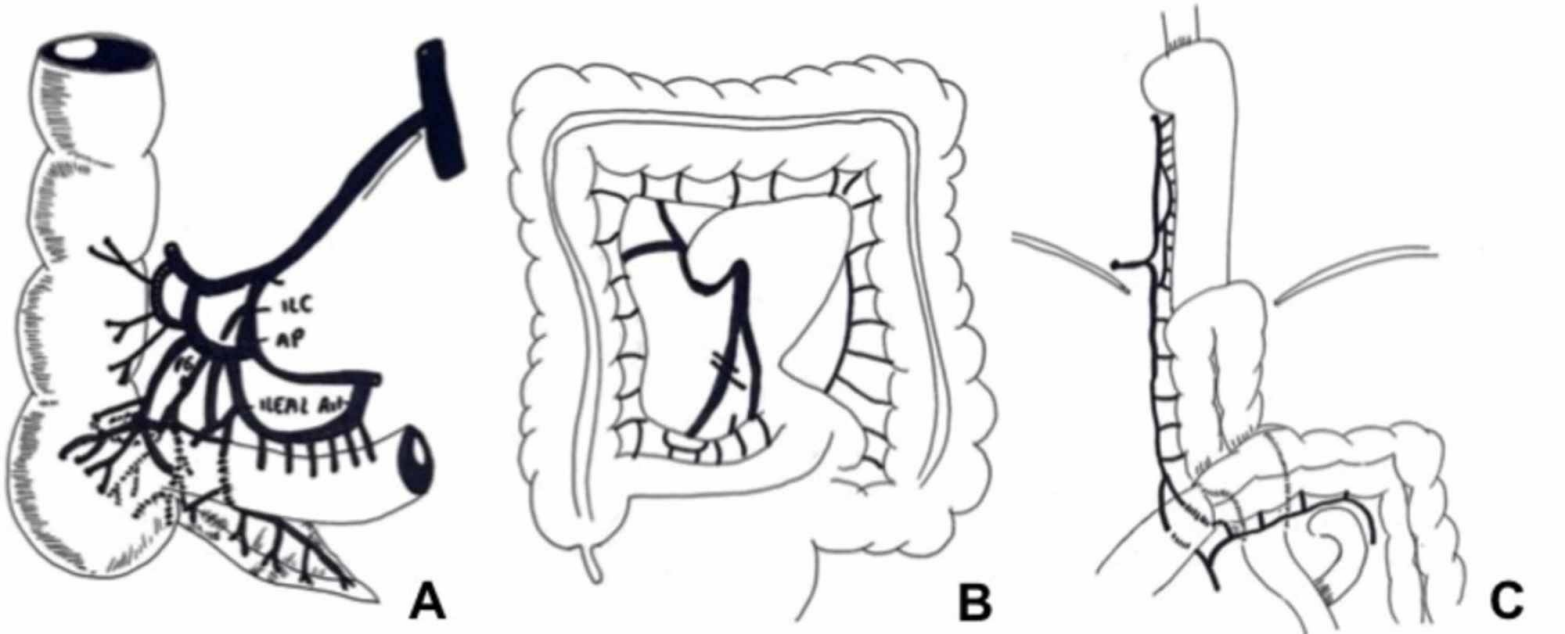
(A) Irrigation diagram of the ileocecal region. (B) Diagram of marginal vascular communications of the main arteries in the ileocolic region. (C) Diagram of esophageal reconstruction showing esophageal-ileal, colojejunal, jejunum-jejunal, and ileo-transverse anastomosis. Adapted from [4].

In August 2018, a new PET-CT confirmed relapse in the distal portion of the esophagus and these new findings were presented at a gastrointestinal surgery meeting, where it was decided to perform a three-way esophagectomy with ileocolonic reconstruction. The procedure began with the patient in a prone position and video-assisted thoracoscopy was used to release pleural-pulmonary adhesions. The esophagus was identified and dissected circumferentially to the thoracic operculum. The procedure continued with the patient in the supine position and affected adhesions were released using laparotomy. The previous anastomosis firmly attached to the pericardium and the prevertebral space were identified and released, and the procedure was continued by left cervicotomy. The cervical esophagus was identified and sectioned. The surgical specimen was removed via transhiatal, and the right colon was released, preserving the right colic artery and marginal veins of the distal ileum. The right colon was raised through the posterior mediastinum to the cervical region via transhiatal and anastomosis of the cervical esophagus to the previously sectioned ileum was performed (Figure 3) [4].

**Figure 4:**
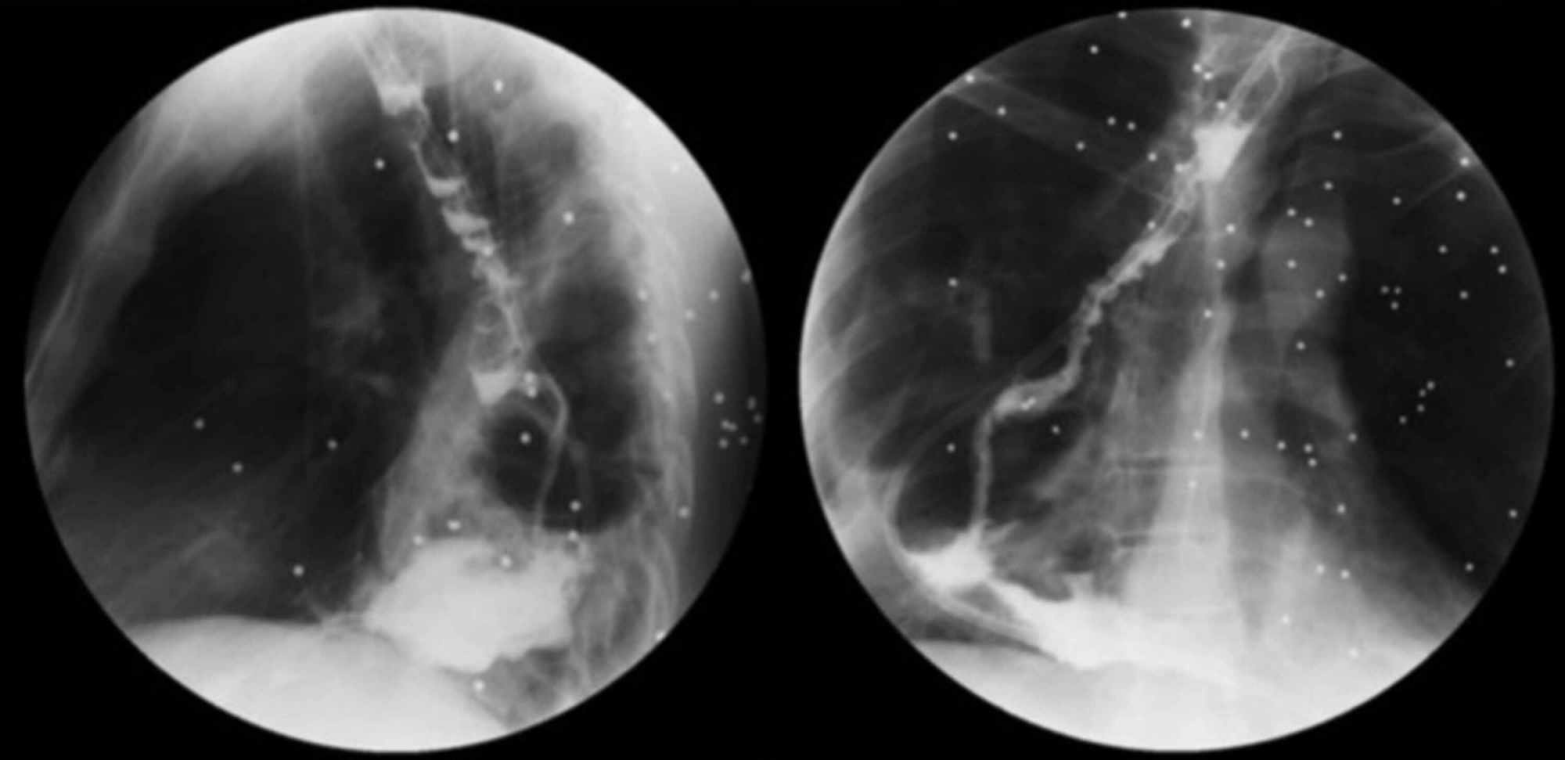
Digestive tract with multiple metallic foreign bodies in the chest, adequate transit of the contrast medium through the cervical anastomosis, ileocecal valve and colojejunal anastomosis

**Figure 5:**
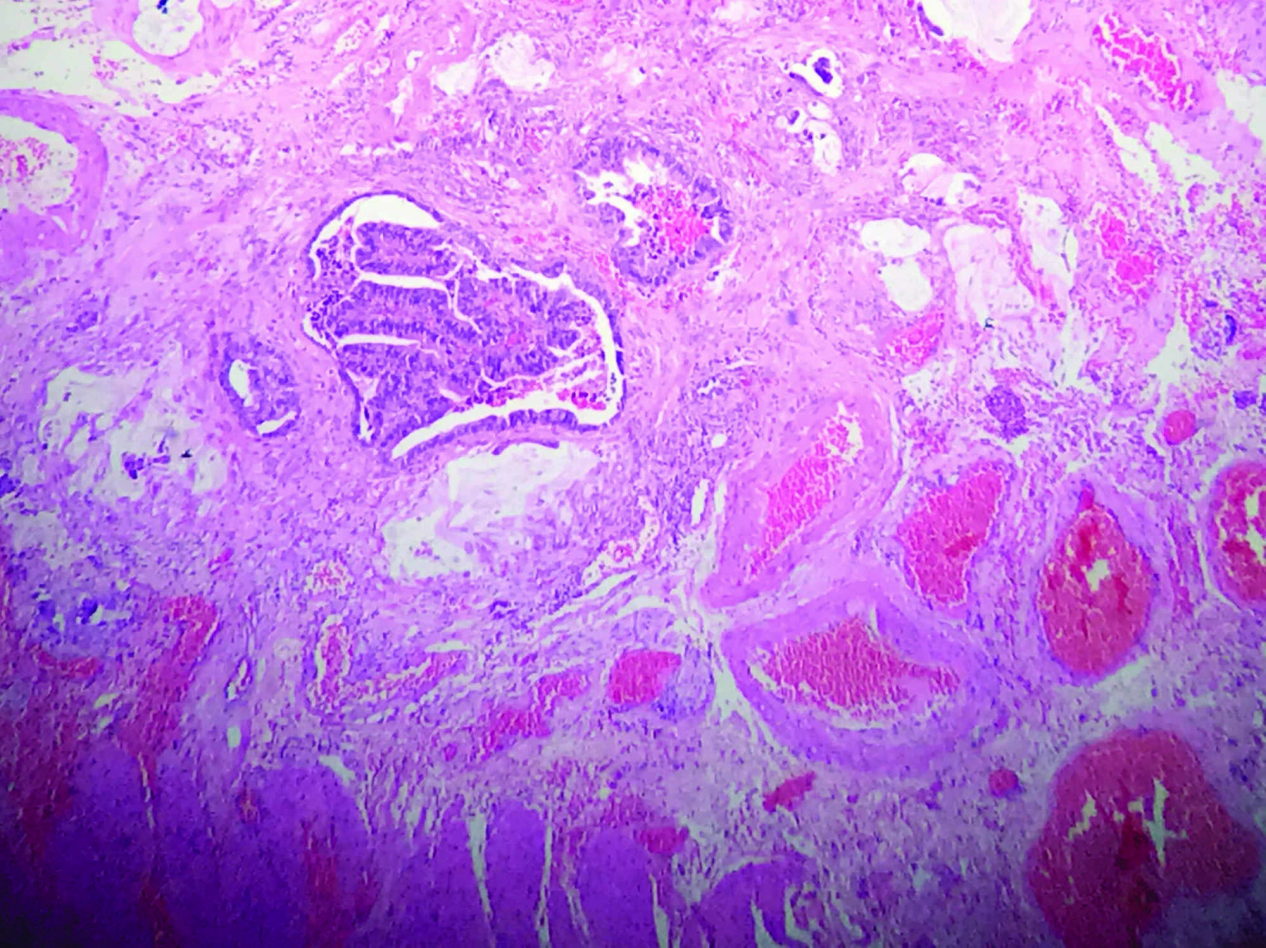
4X Hematoxylin & Eosin (H-E). Esophageal wall with lymphovascular invasion due to adenocarcinoma

The alimentary loop of the jejunum was anastomosed to the ascended colon. The procedure was completed with an ileo-transverse anastomosis and closure of the abdominal cavity. During the postoperative period, there was no evidence of anastomosis leakage in the digestive tract (Figure 4), and surgical pathology reported esophageal-jejunal anastomosis with adenocarcinoma involvement with free section edges (Figure 5).

During the clinical oncology evaluation, it was decided to start capecitabine and oxaliplatin (CLASSIC) for six cycles, which was received without complications. Currently, the patient has no evidence of new relapses.

## Discussion

The incidence of EGJ carcinoma has increased significantly since 1970 in the United States, and similar increases have been reported in Sweden, Canada, and Great Britain [1]. In Colombia, there are no records or incidence figures. According to Globocan 2018, there are 7,419 gastric cancer cases annually and it represents 7.3% of all cancer cases. Furthermore, it is the leading cause of cancer mortality in Colombia with 5,505 deaths per year [5]. Despite aggressive surgical treatment, the prognosis for tumors in this region is poor, with a five-year survival rate of 20%-50% [2,6].

The endoscopic study is the fundamental pillar to define the best treatment for patients with EGJ lesions since defining the exact location predicts patterns of lymph node metastasis [7]. In this initial study, the length of the tumor, the involvement of the circumference, and the location of the squamous-columnar junction are always defined, which vary according to the height of the patient. Within these parameters, the location of the tumor epicenter can be defined and classified according to Siewert and Stein at I, II, or III [8]. In many cases, it is not possible to identify the squamous-columnar junction when a tumor or hiatal hernia is present; in these cases, the start of the gastric folds should be considered an anatomical reference [9]. In addition to endoscopic evaluation, imaging should be done for systematic staging and to determine lymph node involvement. However, there is still a discrepancy between preoperative findings by endoscopy, endosonography, or tomography and the final pathology.

Esophagectomy is indicated in the treatment of EGJ type I tumors using a thoracic and abdominal approach, while gastrectomy and lymphadenectomy with an abdominal or transhiatal approach are sufficient for type III tumors [9]. But for type II EGJ tumors the optimal approach is still being discussed and, according to its characteristics a total transhiatal gastrectomy plus distal esophagectomy can be performed, or an esophagectomy plus proximal gastrectomy, via thoracic and abdominal approach. Or even, in advanced tumors, an esophagectomy could be executed with interposition of the colon or jejunum by three routes [9].

Esophageal involvement greater than 25 mm is a significant preoperative predictor of recurrence, with significantly lower disease-free and overall survival rates compared to those with involvement ≤25 mm (p < 0.001) [10]. The need to achieve adequate surgical exposure for an appropriate oncological resection margin makes the transhiatal abdominal route not the one of choice for patients with EGJ tumors and an esophageal involvement greater than 25 mm. The transthoracic abdominal approach is recommended. The patient presented in this study had a 20-mm compromise of the distal esophagus and therefore an abdominal approach was initially decided on, but the proximal resection margin was positive (with negative esophageal donut), despite a 5-cm resection of the distal esophagus. This reminds us of the importance of an adequate initial localization to offer the patient the best initial therapeutic option and a better overall and disease-free survival rate [10].

Lymph node involvement in all oncological pathologies, as well as in EGJ tumors, is a prognostic factor for overall recurrence and survival rates. These tumors have the particularity that lymphatic drainage pathways include both the abdomen and the mediastinum [7]. In radical surgery, both the primary tumor and lymphatic drainage must be resected, taking into account that 16% of these tumors involve lymph nodes in the lower mediastinum [11]. A study published in 2019 showed that 5.1% of patients with EGJ tumors with esophageal involvement greater than 3 cm had lymph node involvement around the right recurrent laryngeal nerve and in the esophageal stations of the middle mediastinum. If esophageal involvement was greater than 4 cm, more than 10% presented lymph node involvement in the mentioned stations. Therefore, in this study, the recommendation is to perform a subtotal esophagectomy and drainage of the upper and middle mediastinal nodes in patients with EGJ tumors with esophageal involvement greater than 4 cm [12]. Other authors recommend mediastinal lymph node dissection in patients who have from one to eight nodes compromised [9]. The number of resected nodes is controversial. In gastric cancer, a minimum of 15 nodes should be resected according to the AJCC, although some authors suggest that resecting more than 25 or 35 nodes could improve survival [13]. For esophageal cancer, some authors describe better survival rates if more than 21 nodes are resected, but there are no specific studies for EGJ tumors [14].

Positive margins in oncological surgeries for EGJ tumors are presented in up to 35% of cases mainly due to the proximal resection margin, but this incidence lowers to 0% if they are resected more than 6 cm. Subsequent studies have failed to demonstrate an association between proximal margin length and recurrences [9].

EGJ tumor recurrence can occur in 20% to 50% of cases, usually in the first two years of surgery for the primary tumor [15]. There is no consensus on the risk factors associated with the recurrence of these tumors, and reported factors include tumor size, lymph node involvement, lymphovascular invasion, perineural invasion, and poor tumor differentiation [6].

In the described case, the patient presented two recurrences. In the first one, the previous anastomosis was resected via transhiatal and a new esophageal-jejunal anastomosis was performed, but in the second recurrence greater esophageal involvement was evidenced, so it was decided to perform an esophagectomy using the right colon and the distal ileum for reconstruction.

Patients with recurrences have a median survival of 22 months and surgical resection has improved survival in some series, but not in others [15]. In a series of 60 patients with recurrence of gastric cancer and EGJ undergoing surgical reintervention as reported by Badgwell et al., resection with curative intent was achieved in 29 patients (52%), 13 patients were treated with total gastrectomy, nine with esophagectomy, three with esophagectomy and six required reconstructions with colonic or jejunal interposition. The overall survival rates were 72%, 38%, and 28%, at one, three, and five years, respectively, in R0 patients. Morbidity was 52% and postoperative mortality (3%) was present. Overall survival rates were higher in patients who were resected compared with patients who had unresectable disease (25.8 months vs. six months) [15].

Traditionally, the stomach has been the first choice to replace the esophagus due to the rich submucosal vasculature, easy mobilization, and the need for just one anastomosis [16]. In our patient, this organ could not be used because he already had a history of gastrectomy.

There are other alternatives for cases where the stomach cannot be used. In the case of our patient, during the resection of the first local recurrence, the distal esophagus had to be replaced with the jejunum. This organ can also be used as an interposition between the esophagus and the stomach as a free flap and has the advantages that it is an abundant organ, does not require preparation, is usually disease-free, has a diameter similar to the esophagus, has intrinsic peristalsis, and in general, its length does not change over time [17].

During the second recurrence of adenocarcinoma in esophageal anastomosis with the jejunum, the colon was used to replace the esophagus. The left colon is easy to mobilize and adequate irrigation is provided by the left colic and middle colic veins. Furthermore, mucus production functions as an antireflux barrier, and the isoperistaltic mechanism helps transit of food bolus; however, extensive mobilization and three anastomoses are required, which could increase morbidity [3].

The right colon that was used in reconstruction has a thinner wall when compared to the left colon, is more voluminous, and peristalsis is slower. Its main disadvantage is the absence of a marginal artery in a third of cases, making it necessary to preserve the arterial arches of the ileocecal area, obtaining a long ileum segment (up to 20 cm) and thus ensuring suitable blood flow and length to replace the thoracic and cervical esophagus [4]. In our case, we preferred to use the right colon and distal ileum because we had a good vascular supply, an easy ascent through the mediastinum for a cervical anastomosis, and an ileum caliber more suitable for anastomosis with the cervical esophagus. In addition, moving the transverse colon with multiple adhesions from previous surgeries was avoided.

A case series published in 2012 in which esophageal reconstruction with ileocolic interposition was performed with six patients did not report mortality at 30 days, one patient died of pneumonia within 90 days, and there were no anastomotic leaks or graft necrosis [4].

Finally, it was decided to do a hybrid technique with thoracoscopy and laparotomy because we wanted to give the patient the opportunity to have the advantages of minimally invasive surgery [18]. Since the patient had multiple previous surgeries, the abdominal procedure was performed using an open technique and the thoracic procedure using thoracoscopy. In Colombia and Latin America, there are multiple series of patients undergoing minimally invasive or hybrid esophagectomy [19], and only a few case reports have described patients who have been treated with a right colon ascent with a hybrid technique [20].

## Conclusions

There are multiple consequences of an inadequate classification of esophagogastric junction tumors. Mainly, it involves the erroneous choice of surgical approach, and the oncologic treatment, such as the temporal relationship between chemotherapy, radiotherapy, and surgery that depends on a clear topographic understanding and accurate classification of these tumors. Perioperative chemotherapy is the choice for Siewert III tumors and neoadjuvant chemoradiation therapy, such as CROSS, is the choice in Siewert I and II tumors. Finally, adequate initial decisions could reduce the risk of relapse, which could lead to increased morbidity and mortality, and thus avoid the need for complex esophageal reconstructions such as in the reported case. Surgical resection should always be considered in patients with isolated local recurrences since it improves overall survival rates. Gastrointestinal and oncological surgeons should be familiar with the different techniques to treat recurrent EGJ cancer so they can handle complex cases such as the patient described in this case report, in which it was finally necessary to use the right colon and distal ileum for esophageal reconstruction.
